# Gut Microbiota Signatures in Tumor, Para-Cancerous, Normal Mucosa, and Feces in Colorectal Cancer Patients

**DOI:** 10.3389/fcell.2022.916961

**Published:** 2022-06-02

**Authors:** Yanmin Li, Hong Cao, Bojian Fei, Qizhong Gao, Wanya Yi, Weifeng Han, Chuanqing Bao, Jianmin Xu, Wei Zhao, Feng Zhang

**Affiliations:** ^1^ State Key Laboratory of Food Science and Technology, School of Food Science and Technology, Jiangnan University, Wuxi, China; ^2^ Department of Nutrition, Affiliated Hospital of Jiangnan University, Wuxi, China; ^3^ Department of Gastrointestinal Surgery, Affiliated Hospital of Jiangnan University, Wuxi, China; ^4^ Chinese Society of Nutritional Oncology, Beijing, China

**Keywords:** colorectal cancer, Fusobacterium, 16S rRNA, gut microbiota, fecal occult blood test

## Abstract

**Background:** Association studies have linked microbiome alterations with colorectal cancer (CRC). However, differences in tumor, para-cancerous, normal mucosal, and fecal microbiota remain to be strengthened.

**Methods:** We performed a study on the ecologically rich and taxonomically diverse of gut microbiota using three types of colorectal mucosa (tumor mucosa, para-cancerous mucosa, normal mucosa) and feces from 98 CRC patients. Additionally, we profiled the microbiota in the fecal occult blood test (FOBT) positive and negative groups at different sampling sites.

**Results:** We found striking variations between tumor mucosal microbiota and normal mucosal microbiota. However, there was no significant difference between tumor and para-cancerous mucosal microbiota, as well as between para-cancerous and normal mucosal microbiota, revealing that the para-cancerous mucosal microbiota was a transitional state between the tumor and normal mucosal microbiota. And the substantial shifts in the fecal microbiota compared to mucosal microbiota indicated the risk of using fecal microbiota to define mucosal microbiota. A strong correlation between FOBT positive and *Fusobacterium* was discovered, indicating this adherent-invasive genus was closely related to intestinal bleeding. Furthermore, we identified six key genera, including *Fusobacterium*, *Gemella*, *Campylobacter*, *Peptostreptococcus*, *Alloprevotella*, and *Parvimonas*, which appear to be consistently over-represented in tumor mucosa compared to normal mucosa and/or in mucosa compared to feces.

**Conclusion:** Compositional alterations in the microbiota existed in three types of colorectal mucosa and feces in CRC patients. Six key genera may contribute to the topographic variances in the microbiota of tumor-bearing colorectum.

## Introduction

Worldwide, colorectal cancer (CRC) ranked third for incidence and second for mortality as dietary changes. According to the Global Cancer Statistics, about 935,000 people were estimated to die of CRC in 2020 (Sung et al., 2021). Thus, there is an urgent need for early detection and prevention of CRC. Several independent factors that affect the risk of CRC have been identified in epidemiologic research, including lack of physical activity, obesity, and high consumption of red and processed meat (Kerr et al., 2017).

Gut microbiota exhibits substantial impacts on the pathogenesis of several cancers, the most notable being *Helicobacter pylori* and gastric cancer, and some gastric lymphomas (Amieva and Peek, 2016). Accumulating evidence demonstrated that the gut microbiota plays a vital role in the etiology of CRC (Tilg et al., 2018). The potential roles of particular pathogenic bacteria such as *Fusobacterium nucleatum*, *Porphyromonas*, *Campylobacter*, and *Peptostreptococcus* in developing CRC through promoting inflammatory interactions with host cells have been reported recently (Castellarin et al., 2012; Kostic et al., 2012; Kostic et al., 2013; Rubinstein et al., 2013; Long et al., 2019; Okuda et al., 2021). In addition to the direct effects of certain bacteria on tissues, broader microbial communities may modify the risk of CRC through multiple mechanisms such as competitive exclusion. Gut microbial metabolites, such as short-chain fatty acids (SCFAs) and bile acids, have a considerable influence on CRC prevention and predisposition (Donohoe et al., 2014; Louis et al., 2014). Thus, the utilization of gut microbiota sequencing for exploring the pathogenesis, early diagnosis, and prevention of CRC has attracted tremendous attention.

Of note, the findings of previous studies have not uniform nor conclusive. These variances may be related to differences in primary tumor sites (Dejea et al., 2014; Gao et al., 2015; Flemer et al., 2017; Phipps et al., 2021), sampling sites (mucosa *vs.* feces) (Warren et al., 2013; Nakatsu et al., 2015; Flemer et al., 2017), and stages of disease (Yachida et al., 2019), which have posed opportunities and challenges to the diagnosis and treatment of CRC. Evidence from recent years has shown the substantial shifts in the fecal microbiota of CRC patients compared to that of healthy individuals, implying that fecal microbiota has possible diagnostic potential for CRC (Wirbel et al., 2019). Despite the obvious microbial differences between CRC patients and healthy individual were observed, the tumor-associated microbiota and non-tumor-associated microbiota within the same individual did not differ significantly (Flemer et al., 2017). Notably, one study described the relative abundance of *Roseburia* in feces of CRC patients was lower than that in healthy individuals, in contrast to sequencing results in the mucosa, further demonstrating discrepancies between fecal and mucosal microbiota in CRC patients (Chen et al., 2013; Mira-Pascual et al., 2015). Recent large-cohort sequencing data revealed that shifts in the fecal microbiome and metabolome occurred in the early stages of CRC, which is of potential aetiological and diagnostic significance (Yachida et al., 2019).

Fecal occult blood test (FOBT) is a convenient and cheap non-invasive tool for screening advanced carcinomas by detecting traces of blood in feces released from colorectal lesions and is one of the most widely used first-line approaches for CRC. The utilization of FOBT has been shown to decrease mortality from CRC in several large-scale randomized trials (Mandel et al., 1993; Kewenter et al., 1994; Hardcastle et al., 1996). According to multiple international and national guidelines recommendations, individuals at average risk should undergo organized screening for advanced adenoma and CRC (Arbyn et al., 2010). Among the existing FOBTs, the fecal immunochemical test (FIT) seems superior to the high-resolution guaiacol FOBT in terms of detection rate and positive predictive value for CRC (Argiles et al., 2020). It is worth noting that the combined use of FOBT and metagenomic CRC detection could improve diagnostic sensitivity for CRC while retaining respective specificity (Zeller et al., 2014; Yuan et al., 2021).

We conducted a study on the ecologically rich and taxonomically diverse of colorectal microbiota using three types of colorectal mucosa and feces from individuals with CRC. Additionally, we profiled the microbiota in the FOBT positive and negative groups at different sampling sites. Our data revealed compositional alterations in the microbiota existed in three types of colorectal mucosa and feces in CRC patients, which represents a step toward characterizing microbial consortia in CRC carcinogenesis.

## Materials and Methods

### Patient Recruitment

Ethical approval was granted by the Ethics Committee of the Affiliated Hospital of Jiangnan University, Wuxi, China (LS2021026), and was registered at the Chinese Clinical Trial Registry (ChiCTR2100046237). Patients were recruited from May 2021 to September 2021. Patients scheduled for colorectal resection were recruited to this study. Patients under chemotherapy or radiotherapy, those who were treated with antibiotics, anticoagulants, or probiotics/prebiotics in 4 weeks prior to surgery, and those who had other gastrointestinal tract diseases such as diverticula, inflammatory bowel disease and acute gastroenteritis, were all excluded. Written informed consents were obtained from participants who agreed to participate in this study.

### Sample Collection

Mucosal tissues were collected from CRC patients during surgery from three different sites: 1) tumor mucosa, 2) para-carcinoma mucosa (2 cm away from the tumor), and 3) normal mucosa (near the surgical resection margins; usually 10–30 cm away from the tumor). Each tissue was sliced into one cubic centimeter pieces and placed in cryopreserved tubes. The tissues were preserved in the -80°C refrigerator after being frozen in liquid nitrogen. Before preoperative bowel preparation, two fecal specimens from each patient were obtained in the morning and transferred to the laboratory’s -80 °C refrigerator within 30 min.

### DNA Extraction and 16S rRNA Amplicon Sequencing

Microbial community genomic DNA was extracted from 200 mg fecal samples or 300 mg tissue samples using DNA kit (Omega Bio-tek, Norcross, GA, United States ). 1% agarose gel electrophoresis was used to check the DNA integrity, and NanoDrop 2000 UV-vis spectrophotometer (Thermo Scientific, United States ) was used to detect the DNA concentration and purity. The hypervariable region V3-V4 of the microbial 16S rRNA gene was amplified by an ABI GeneAmp® 9,700 polymerase chain reaction (PCR) thermocycler (ABI, CA, United States ) using the following primer pair: 338F, 5′-ACT​CCT​ACG​GGA​GGC​AGC​AG-3′, and 806R, 5′-GGACTACHVGGGTWTCTAAT-3'. The protocol of PCR thermocycler was as follows: 3 min at 95°C followed by 29 cycles of 30 s at 95°C, 30 s at 55°C and 45 s at 72°C, and a final 5 min at 72°C. PCR reactions were performed in three replicates. PCR amplicons were extracted using 2% agarose gel, purified using AxyPrep DNA Gel Extraction Kit (Axygen Biosciences, Union City, CA, United States ), and quantified using Qubit4.0 (Thermo Fisher, United States ). Purified amplicons were pooled in equimolar and paired-end sequenced on the Illumina MiSeq PE300 platform (Illumina, San Diego, United States ) according to the standard protocols by HonSunBio Technology Co. Ltd (Shanghai, China).

### Bioinformatics Analysis

After sequencing, the raw 16S rRNA gene sequencing reads were quality-filtered by fastp (version 0.20.0) (Chen et al., 2018) and merged by FLASH (version 1.2.11) (Magoc and Salzberg, 2011). Operational taxonomic units (OTUs) were clustered with a 97% similarity cutoff using UPARSE platform (version 7.1) (Edgar, 2013), and chimeric sequences were identified and removed. The taxonomy of each OTU representative sequence was analyzed by the RDP Classifier (version 2.2) (Wang et al., 2007) against the Silva database (SSU138) with a confidence threshold of 0.7. α-diversity was estimated using the ACE, Chao1, Shannon, and Simpson index by mothur (version 1.30.1). Principal coordinates analysis (PCoA) based on bray-curtis matrices with statistical significance determined by permutational multivariate analysis of variance (PERMANOVA) was conducted using functions “pcoa”, and “adonis” from the R package vegan to assess the differences in β-diversity between groups. For comparing the relative abundance of different taxa between groups, linear discriminant analysis (LDA) was conducted on the LEfSe with a *p-*value < 0.05 for the Kruskal–Wallis test and a size-effect threshold of 2.0 on the logarithmic LDA score.

### Fecal Occult Blood Test

Fecal samples were examined using hemoglobin/transferrin joint detection test kit (colloidal gold immune chromatography, W.H.P.M.) according to the instructions of the manufacturer. Feces were collected at multiple points and were mixed thoroughly with deionized water. The prepared specimen was added to the specimen area, and test results were visually interpreted by lines in the test line area and control line area after 2 min. The cutoff value for positive FOBT was 10 ng/ml.

### Dietary Record

Dietary information was estimated using a 24-h dietary record questionnaire of the day before feces collection.

### Statistical Analysis

R software (version 3.6.3) and associated packages (ggplot2, ggforce, ggsci, RColorBrewer, dplyr, reshape2, rstatix, and vegan), SPSS (version 22.0), and GraphPad Prism (version 9.2) were used to analyze data and draw figures. Continuous data were expressed as mean ± standard error of the mean (SEM), and categorical data were expressed as the number of cases (n) and percentage (%). Mann-Whitney U test for independent samples was applied to compare alpha diversity indexes and abundance of key genera between groups. Results were regarded as statistically significant if *p-*values < 0.05.

## Results

### Clinicopathological Status of Patients

In total, 98 participants scheduled for colorectal resection were recruited to this study, including 58 males and 40 females. For 77 individuals, three types of mucosal samples, including tumor mucosa (T), para-carcinoma mucosa (P), and normal mucosa (N), as well as fecal samples (F), were collected. For 16 individuals, only three types of mucosal samples were collected. For five individuals, only fecal samples were collected. The average age of participants was 64.77 ± 0.92 years. According to the tumor-node-metastasis (TNM) classification of malignant tumors, all participants with CRC were divided into I-IV stages: 10 as stage I, 51 as stage II, 30 as stage III, and 7 as stage IV. In addition, the number of left and right segments collected was 59 and 39, respectively. The demographic data, dietary information, and clinical data of CRC patients were shown in Table 1.

**TABLE 1:** Patient characteristics.

Characteristics	Enrolled patients (n = 98)
Age, mean ± SEM, y	64.77 ± 0.92
Gender	
Male (%)	58 (59.18%)
Female (%)	40 (40.82%)
Height, mean ± SEM, m	1.64 ± 0.01
Weight, mean ± SEM, kg	63.92 ± 1.04
BMI, mean ± SEM, kg/m^2^	23.79 ± 0.31
Dietary intake	
Energy intake, mean ± SEM, kcal	420.22 ± 32.40
Protein intake, mean ± SEM, g	16.79 ± 1.42
Fat intake, mean ± SEM, g	10.03 ± 1.79
Carbohydrate intake, mean ± SEM, g	65.93 ± 4.36
Fiber intake, mean ± SEM, g	1.53 ± 0.27
ECOG performance status	
0 (%)	61 (62.25%)
1 (%)	37 (37.75%)
TNM stage	
Ⅰ (%)	10 (10.20%)
Ⅱ (%)	51 (52.04%)
Ⅲ (%)	30 (30.61%)
Ⅳ (%)	7 (7.14%)
Tumor location	
Left-sided (%)	59 (60.20%)
Right-sided (%)	39 (39.80%)
Tumor markers	
CEA, mean ± SEM, ng/mL	18.99 ± 6.49
AFP, mean ± SEM, ng/mL	2.70 ± 0.14
CA19-9, mean ± SEM, U/mL	46.15 ± 13.28
Size of tumor, mean ± SEM, cm	4.47 ± 0.19
Degree of tumor differentiation	
Well-differentiated (%)	11 (11.22%)
Moderately-differentiated (%)	81 (82.65%)
Poorly differentiated (%)	6 (6.12%)

### Microbial Profiles of the Samples

A total of 17,722,655 valid sequences (416 bp on average) were created from 361 specimens, and a total of 2,227 OTUs were identified after data filtration. The Good’s coverage of each sample was >99%, implying that the 16S rRNA sequences identified could represent the majority of taxa present in the specimens.

### Microbiota Variation in Different Sampling Sites

**FIGURE 1:**
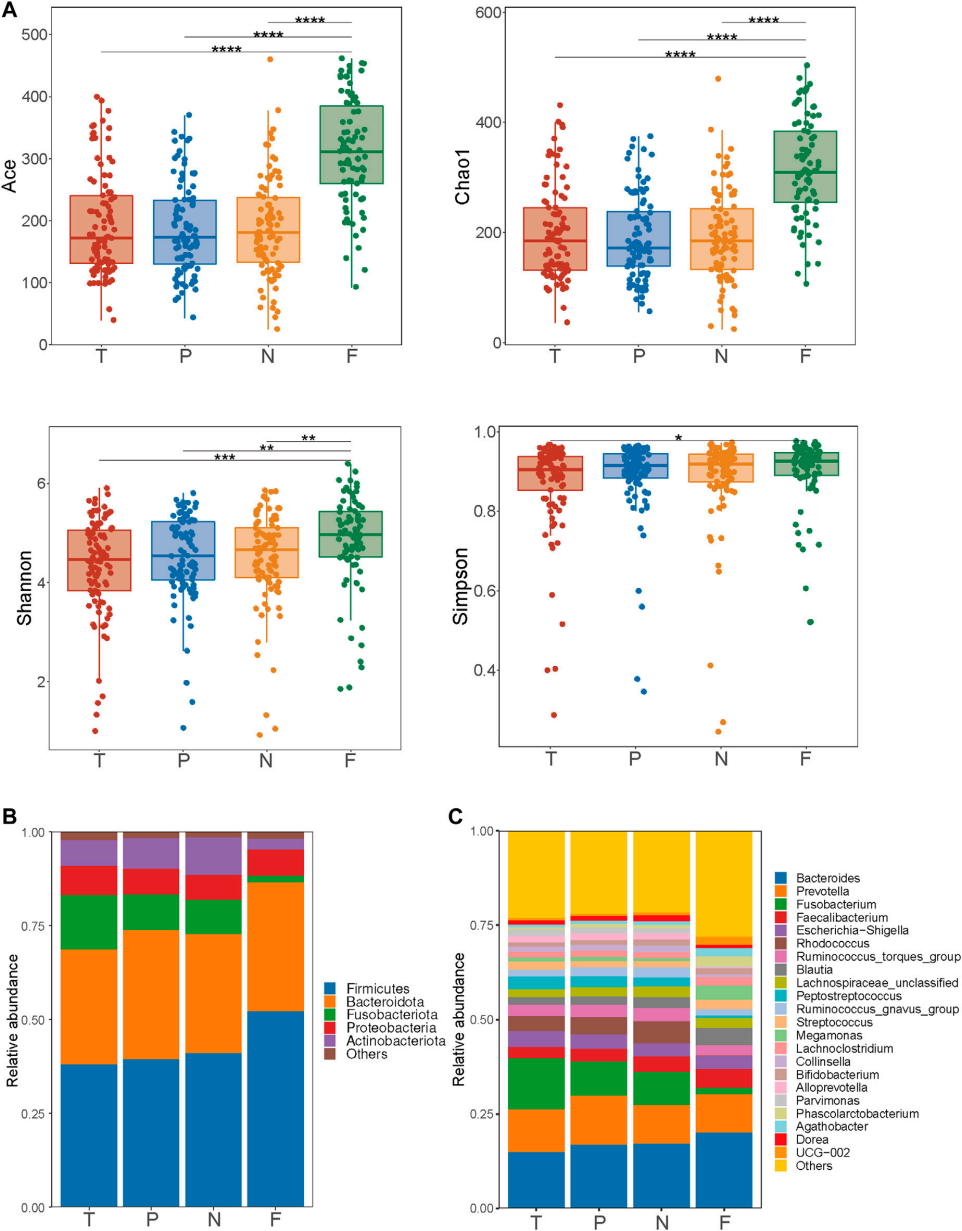
Analysis of α diversity indexes and relative abundance (%) of microbiota at different levels in tumor mucosa (T), para-cancerous mucosa (P), normal mucosa (N), and feces (F). **(A)** Comparison of α diversity indexes (ACE, Chao1, Shannon, and Simpson) between different mucosal samples and between mucosa and feces. **(B)** Relative abundance (%) of phyla in mucosa and feces samples. **(C)** Relative abundance (%) of genera in mucosa and feces samples. *p < 0.05, **p < 0.01, ***p < 0.001, ****p < 0.0001.

Bacterial communities from the triplet-paired mucosal and fecal samples of CRC patients were analyzed. The number of samples in each group was as follows: T group (*n* = 93), P group (*n* = 93), N group (*n* = 93), and F group (*n* = 77). Microbial richness and diversity, which were estimated by ACE, Chao1, Shannon, and Simpson indexes, were not statistically different among the three types of mucosa samples (Figure 1A). Nevertheless, ACE and Chao1 indexes showed significant increases in feces compared to three types of mucosa samples (*p* < 0.001).

We further observed microbial communities of tumor, para-cancerous and normal mucosa at different levels. At the phylum level (Figure 1B), the five dominant phyla in each group were Firmicutes, Bacteroidota, Fusobacteriota, Actinobacteriota, and Proteobacteria. At the genus level (Figure 1C), *Bacteroides* (15.01%, 16.90%, 17.23%, and 20.18%, respectively) showed the highest proportions in the tumor mucosa, para-cancerous mucosa, normal mucosa, and fecal samples. Within para-cancerous mucosa, normal mucosa, and fecal samples microbiota, the next most abundant genus identified was *Prevotella* (12.98%, 10.17 %, and 10.01%, respectively). *Fusobacterium*, widely reported to play a vital role in promoting colorectal carcinogenesis (Kaplan et al., 2009; Kaplan et al., 2010), accounted for 13.52% of tumor mucosal microbiota, 9.12% of para-cancerous mucosal microbiota, 8.87% of normal mucosa, and 1.75% of fecal microbiota.

**FIGURE 2:**
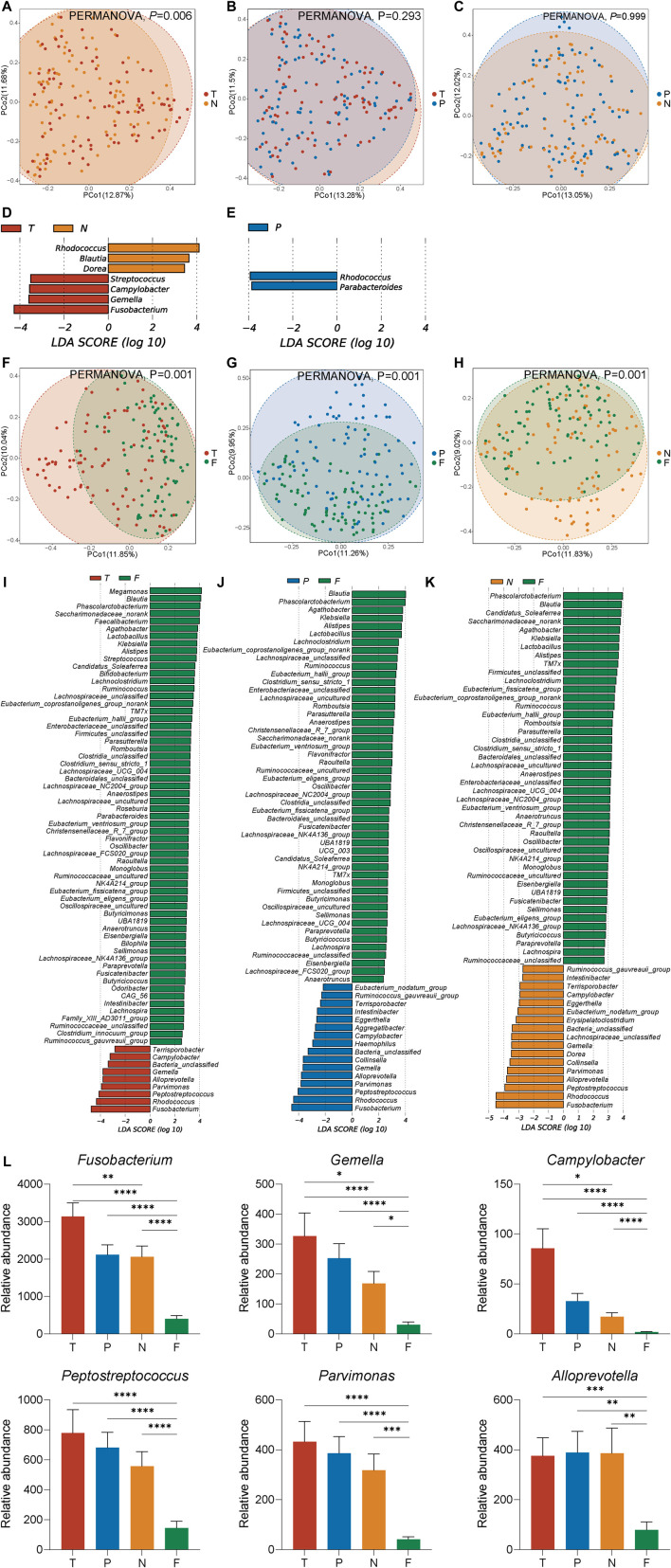
Microbial community ecology for tumor mucosa (T), para-cancerous mucosa (P), normal mucosa (N), and feces (F) **(A–C)** Comparison of β diversity between different mucosal samples **(F–H)** Comparison of β diversity between mucosa and feces **(D,E)** Histograms of LDA scores (>2.0) for differentially abundant genera between different mucosal samples **(I–K)** Histograms of LDA scores (>2.0) for differentially abundant genera between mucosa and feces **(L)** Relative abundance of key genera in four groups selected by LEfSe including *Fusobacterium*, *Gemella*, *Campylobacter*, *Peptostreptococcus*, *Alloprevotella*, and *Parvimonas*. *p < 0.05, **p < 0.01, ***p < 0.001, ****p < 0.0001.

PCoA based on bray-curtis matrices indicated that mucosal microbiota of tumor tissues differs significantly from normal tissues (*p* < 0.01, Figure 2A). There was no statistical difference in the microbiome clusters between tumor and para-cancerous mucosa (*p* = 0.293, Figure 2B), as well as between para-cancerous and normal mucosa (*p* = 0.999, Figure 2C). And significant separations in microbial community compositions between three types of mucosa and feces were observed (*p* < 0.001, Figure 2F–H).

Next, we used LEfSe analysis to identify significant bacterial taxa between different sampling sites. In the comparison of tumor mucosal microbiota and normal mucosal microbiota (Figure 2D), *Fusobacterium*, *Gemella*, *Campylobacter*, and *Streptococcus* were the key biomarkers for tumor mucosa, while *Rhodococcus*, *Blautia*, and *Dorea* were the key biomarkers for normal mucosal microbiota. In the comparison of tumor mucosal microbiota and para-cancerous mucosal microbiota (Figure 2E), *Rhodococcus* and *Parabacteroides* were found over-represented in para-cancerous mucosal microbiota. Consistent with the above PCoA result, there was no genus screened out in the comparison of para-cancerous mucosal microbiota and normal mucosal microbiota. In the comparison of tumor mucosal microbiota and fecal microbiota (Figure 2I), *Fusobacterium*, *Rhodococcus*, *Peptostreptococcu*s, *Parvimonas*, *Alloprevotella*, *Gemella*, *Campylobacter*, and *Terrisporobacter* exhibited more enriched in tumor mucosa, while *Megamonas*, *Blautia*, *Phascolarctobacterium*, Saccharimonadaceae*_norank*, *Faecalibacterium* were the key biomarkers fecal microbiota. In the comparison of para-cancerous mucosal microbiota and fecal microbiota (Figure 2J), *Fusobacterium*, *Rhodococcus*, *Peptostreptococcus*, *Parvimonas*, *Alloprevotella*, *Gemella*, *Collinsella*, *Haemophilus*, *Campylobacter*, *Aggregatibacter*, *Eggerthella*, *Intestinibacter*, *Terrisporobacter*, *Ruminococcus_gauvreauii_group*, *Eubacterium_nodatum_group* showed higher abundance in para-cancerous mucosa, while *Blautia*, *Phascolarctobacterium*, *Agathobacter*, *Klebsiella*, and *Alistipes* were the key biomarkers for fecal microbiota. In the comparison of normal mucosal microbiota and fecal microbiota (Figure 2K), *Fusobacterium*, *Rhodococcus*, *Peptostreptococcus*, *Alloprevotella*, *Parvimonas*, *Collinsella*, *Dorea*, *Gemella*, Lachnospiraceae*_unclassified*, *Erysipelatoclostridium*, *Eubacterium_nodatum_group*, *Eggerthella*, *Campylobacter*, *Terrisporobacter*, *Intestinibacter*, and *Ruminococcus_gauvreauii_group* showed a higher abundance in normal mucosa, while *Phascolarctobacterium*, *Blautia*, *Candidatus_Soleaferrea*, Saccharimonadaceae*_norank*, and *Agathobacter* were the key biomarkers for fecal microbiota.

Of note, we identified six key genera, including *Fusobacterium*, *Gemella*, *Campylobacter*, *Peptostreptococcus*, *Alloprevotella*, and *Parvimonas*, as common biomarkers for mucosal microbiota compared to fecal microbiota, and *Fusobacterium*, *Gemella*, and *Campylobacter* as common biomarkers for tumor mucosal microbiota compared to normal mucosal microbiota. Histograms (Figure 2L) showed that the relative abundance of *Fusobacterium*, *Gemella*, *Campylobacter*, *Peptostreptococcus*, and *Parvimonas* steadily decreased along with the tumor, para-cancerous, normal mucosa to feces. Nevertheless, the relative abundance of *Alloprevotella* in three types of mucosa samples was indistinguishable.

### Microbiota Variation Between Positive FOBT and Negative FOBT

To explore the potential relationship between FOBT and gut microbiota, samples from different sampling sites were divided into positive groups and negative groups according to FOBT results, and differences in gut microbiota between groups were compared. Of the 77 fecal samples, 37 were FOBT positive, and 40 were FOBT negative. The number of samples in each group was as follows: T_Positive (*n* = 37), T_Negative (*n* = 40), P_Positive (*n* = 37), P_Negative (*n* = 40), N_Positive (*n* = 37), N_Negative (*n* = 40), F_Positive (*n* = 37), F_Negative (*n* = 40).

Diet is known to have an effect on the fecal microbiota. Dietary intake was assessed with a 24-h dietary record the day before feces collection. The energy, protein, fat, carbohydrate, and fiber intake between the FOBT positive and FOBT negative patients was not significantly different (*p* > 0.05, Supplementary Figure 1).

Four α-diversity indexes, including ACE, Chao1, Shannon, and Simpson indexes, were not statistically different between the positive and negative groups at different sampling sites (Supplementary Figure 2A).

**FIGURE 3:**
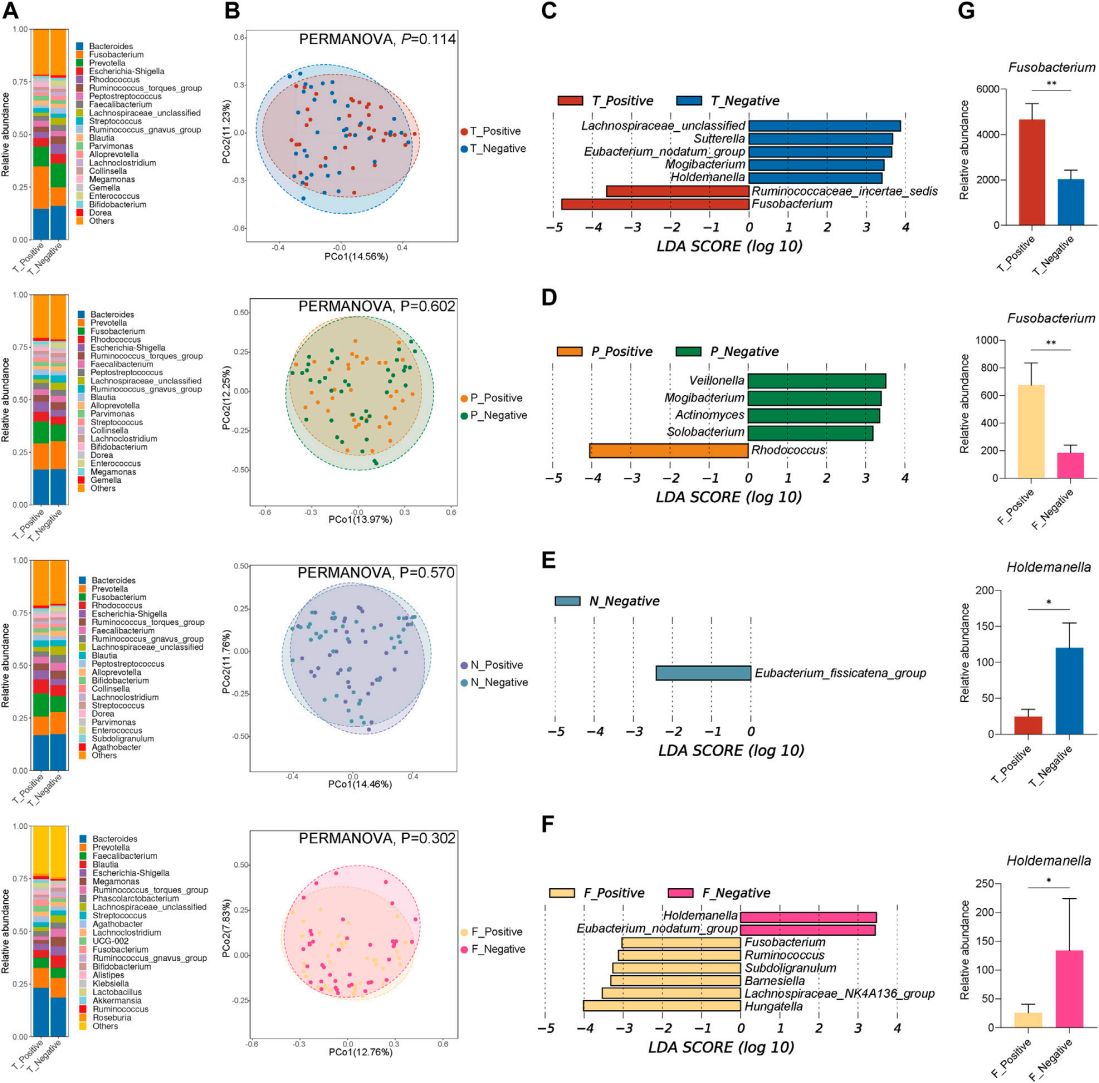
Microbial community ecology differs between the FOBT positive and FOBT negative groups at tumor mucosa (T), para-cancerous mucosa (P), normal mucosa (N), and feces (F) sites. **(A)** Relative abundance (%) of genera in the FOBT positive and FOBT negative groups. **(B)** Comparison of β diversity using PCoA analysis based on bray-curtis distance with statistical significance determined by PERMANOVA between the FOBT positive and FOBT negative groups at different sampling sites **(C–F)** Histograms of LDA scores (>2.0) for differentially abundant genera between the FOBT positive and FOBT negative groups at different sampling sites. **(G)** Relative abundance of *Fusobacterium* and *Holdemanella* in the FOBT positive and FOBT negative groups of tumor mucosa and feces. *p < 0.05, **p < 0.01, ***p < 0.001, ****p < 0.0001.

The compositions of bacterial communities in each group were investigated. At the phylum level (Supplementary Figure 2B), the dominant phyla in each group were Firmicutes, Bacteroidota, Fusobacteriota, Actinobacteriota, and Proteobacteria. At the genus level (Figure 3A), *Bacteroides*, *Prevotella*, and *Fusobacterium* were major contributors to the positive and negative groups at different sampling sites. In the para-cancerous mucosa, the microbial compositions of dominant genera between the positive and negative groups were similar. Nevertheless, the microbial compositions of dominant genera between the positive and negative groups in tumor mucosa and fecal samples were quite different. The relative abundance of *Fusobacterium* in T_Positive group (20.08%) was higher than T_Negative group (8.75%). A similar difference of *Fusobacterium* abundance was discovered in feces between patients with FOBT positive and FOBT negative (2.91% in F_Positive group *vs.* 0.80% in F_Negative group). The dissimilarity of microbiota structural diversities between the positive and negative groups at different sampling sites was assessed by PCoA, revealing that there was no significant difference (Figure 3B).

We next studied variations in the prevalence of key genera identified by LEfSe analysis between positive and negative groups at four sampling sites. For tumor microbiota (Figure 3C), *Fusobacterium* and Ruminococcaceae*_incertae_sedis* were key biomarkers for the T_Positive group, while Lachnospiraceae*_unclassified*, *Sutterella*, *Eubacterium_nodatum_group*, *Mogibacterium*, and *Holdemanella* exhibited more enriched in the T_Negative group. For para-cancerous microbiota (Figure 3D), *Rhodococcus* was the only key biomarker for the P_Positive group, while *Actinomyces*, *Mogibacterium*, *Veillonella*, and *Solobacterium* were key biomarkers for the P_Negative group. For normal microbiota (Figure 3E), Eubacterium_fissicatena_group was the only key biomarker for the N_Negative group. For fecal microbiota (Figure 3F), *Fusobacterium*, *Ruminococcus*, *Subdoligranulum*, *Barnesiella*, Lachnospiraceae*_NK4A136_group*, and *Hungatella* were key biomarkers for the F_Positive group, while *Eubacterium_nodatum_group* and *Holdemanella* exhibited more enriched in the F_Negative group. It is noteworthy that *Fusobacterium* was the common biomarker for the T_Positive group and F_Positive group, and *Holdemanella* was the common biomarker for the T_Negative group and F_Negative group. The relative abundance of *Fusobacterium* and *Holdemanella* in the positive and negative groups from tumor mucosa and feces was shown in Figure 3G.

## Discussion

Variations in tumor, para-cancerous, normal mucosal, and fecal microbiota have posed challenges for deciphering bacterial signatures implicated in CRC carcinogenesis. Here, we investigated the tumor-associated microbial heterogeneity using three types of colorectal mucosa and feces from individuals with CRC. Additionally, we profiled the microbiota in the FOBT positive and negative groups at different sampling sites. We found striking variations between tumor mucosal microbiota and normal mucosal microbiota in CRC participants. Nevertheless, there was no statistical difference between tumor and para-cancerous mucosal microbiota, nor between para-cancerous and normal mucosal microbiota, indicating that the para-cancerous mucosal microbiota was a transitional state between the tumor and normal mucosal microbiota, and the dysbiosis of the para-cancerous mucosal microbiota may precede pathological changes. Data from mucosal sample sequencing were discrepant from those found in fecal samples, implying the risk of using fecal microbiota to define mucosal microbiota.

In particular, *Fusobacterium*, *Gemella*, and *Campylobacter*, as three key biomarkers, appear to be consistently over-represented in tumor mucosa compared to normal mucosa, and in mucosa compared to feces. And *Peptostreptococcus*, *Alloprevotella*, and *Parvimonas* appear to be consistently over-represented in mucosa compared to feces. The relative abundance of *Fusobacterium*, *Gemella*, *Campylobacter*, *Peptostreptococcus*, and *Parvimonas* gradually diminished along with the tumor, para-cancerous, normal mucosa to feces, indicating that the relative abundance of these genera could implicate the degree of mucosal pathological changes and that these genera were more adherent to the tumor mucosa while not being excreted in large quantities in the feces. The relative abundance of these genera in the para-cancerous mucosa was between tumor and normal mucosa, which may partially explain the transitional state of para-cancerous mucosa between the tumor and normal mucosal microbiota. Many of the fecal microbial results reported before may not represent the actual microbial changes in the tumor mucosa. The relative abundance of SCFA-producing genera such as *Blautia* and *Roseburia* in the mucosa, particularly in the tumor mucosa, was lower than in the feces, indicating that SCFAs did not directly exert anti-inflammatory effects on the tumor mucosa. Collectively, mucosal samples, compared to fecal samples, could provide more realistic pictures of the gut microbiota landscape in tumor microenvironments (Osman et al., 2021).

These six genera were previously detected in the oral cavity, with some of them being potential periodontal pathogens, indicating that the links between oral microbiota dysbiosis and gut microbiota dysbiosis may play a role in CRC development. Data from the Human Microbiome Project showed an obvious overlap between the fecal and oral microbiomes, with approximately 45% of taxonomic similarities (Segata et al., 2012). Several studies have identified the enrichment of oral microbiota in colorectal tumors or feces of CRC patients (Kostic et al., 2012; Warren et al., 2013; Nakatsu et al., 2015; Baxter et al., 2016a; Baxter et al., 2016b; Liang et al., 2017; Al-Hassi et al., 2018). According to reports, oral bacteria are presumably disseminated to the gut through swallowing (Nakajima et al., 2015) and bloodstream and systemic circulation (Parahitiyawa et al., 2009).

The aforementioned selected biomarkers were related to epithelial cell adhesion and invasion, biofilm formation, and immunological environment alterations. The integral role of *Fusobacterium* in CRC development has been increasingly elucidated. *Fusobacterium nucleatum* could adhere to the intestinal epithelium by expressing the cell surface proteins FadA, Fap2, and RadD (Wu et al., 2019). RadD further mediates the communications between *Fusobacterium nucleatum* and other bacteria, promoting the formation of microbial biofilms (Kaplan et al., 2009; Kaplan et al., 2010). *Campylobacter* was reported to co-aggregate with *Fusobacterium* (Warren et al., 2013), thus hypothesizing that *Fusobacterium* may act as a bridging microorganism, producing profitable niches for attracting other compatible bacteria and forming biofilms (Koliarakis et al., 2019).

Thin biofilms in the mucosa of healthy individuals are comprised of relatively harmless bacteria, and the majority of these bacteria are commensal species that lack of invasive ability. However, the bacteria detected in biofilms of CRC patients have invasive capabilities. *Fusobacterium nucleatum* has the ability to invade the colorectal mucosa, induce local inflammatory effects, and increase the expressions of interleukin (IL)-6, IL-8, IL-12, transforming growth factor-β (TGF-β), and tumor necrosis factor (TNF), thus possibly promoting CRC (Kostic et al., 2013; McCoy et al., 2013; Saito et al., 2016). *Campylobacter* species such as *Campylobacter concisus*, *Campylobacter rectus*, and *Campylobacter curvus* are harmless in the oral cavity, but they are closely linked to adenocarcinomas of the esophagus and colon (Li et al., 2017). Some *Campylobacter concisus* strains possibly induce the remodeling of cytoskeleton and the disintegration of tight junctions in intestinal epithelial cells by producing zonula occludens toxin, thereby facilitating bacterial translocation and inflammatory responses (Deshpande et al., 2016). *Peptostreptococcus*, a proteolytic pathogen increased in mucosa compared to feces in our data, was reported to be overgrowth in the disrupted mucosal ecosystem, leading to sustained disruption of host colonic proteins and a chronic inflammation state that sustains the production of nutrients for the microbiota, ultimately contributing to CRC tumorigenesis (Louis et al., 2014).

Bacterial biofilms consist of adherent-invasive bacteria that promote the enhancement of gut permeability and the loss of gut barrier function, triggering subsequent inflammatory responses and favoring CRC occurrence (Dejea et al., 2014; Koliarakis et al., 2019; Cueva et al., 2020). And gastrointestinal bleeding, as an aposematic sign for CRC, is related to increased friction with feces and blood vessel rupture caused by tumor proliferation and invasion. In our study, by comparing the differences in the gut microbiota of CRC patients with different FOBT results, we found that *Fusobacterium* emphasized above was enriched in the positive group of tumors and feces compared to the negative group, suggesting that *Fusobacterium* was closely related to intestinal bleeding and reinforcing the idea that *Fusobacterium* was capable of invading the intestinal mucosa (Strauss et al., 2011; Kostic et al., 2013). Furthermore, our data showed that *Holdemanella* was enriched in the negative groups of tumors and feces compared to positive groups. *Holdemanella biformis* and its rodent homolog *Faecalibaculum rodentium*, as SCFAs-producers, were found to play roles in controlling protein acetylation and tumor cell proliferation by suppressing calcineurin/NFATc3 activation (Zagato et al., 2020). The contraction of *Holdemanella* in positive groups may contribute to more severe gastrointestinal hemorrhage. Together, this study revealed a link between positive FOBT and adherent-invasive bacteria.

Limitations to this study are that mucosal tissues and feces from CRC patients were not compared to those from healthy individuals owing to the intestinal mucosa of healthy individuals being more challenging to collect. Although normal mucosa can be collected from healthy individuals during endoscopy, the intestinal environment and sampling methods are not exactly the same as those for mucosa collection during surgery. All participants underwent bowel preparation the day before resection surgery, which is known to change the mucosal microbiota (Jalanka et al., 2015). Although we found a possible association between the gut microbiota and the oral microbiota in CRC patients, the oral samples of these patients were not collected.

In conclusion, we described the topographic variance in the microbiota of tumor-bearing colorectum, which represents a step forward towards defining microbial consortia in CRC tumorigenesis. In the future, metagenomic and metabolomic data gained from fecal/mucosal specimens from CRC patients may in-depth clarify the multifaceted roles of microbial consortia in CRC development and progression, particularly the cause-and-effect relationship between gut microbiota alterations and CRC tumorigenesis. The relationship between certain oral bacteria such as *Gemella* and gut mucosal adhesion and invasion using microbiota transplants, and the correlation between gut microbiota and intestinal bleeding deserve further exploration. There are high therapeutical expectations that good oral hygiene, periodontal therapy, and microecological agents may contribute to preventing intestinal diseases mediated by oral-type microbiota, or that diseases may be potentially prevented by inhibiting the formation of invasive bacteria-mediated biofilms.

## Data Availability

The datasets presented in this study can be found in online repositories. The names of the repository/repositories and accession number(s) can be found below: https://www.ncbi.nlm.nih.gov/bioproject/PRJNA830432.

## Supplementary Material

Supplementary Figure 1 Comparison of dietary intake including energy, protein, fat, carbohydrate, and fiber intake between the FOBT positive and FOBT negative patients.

Supplementary Figure 2 Comparison of α diversity indexes and relative abundance (%) of microbiota at phylum level between the FOBT positive and FOBT negative groups at tumor mucosa (T), para-cancerous mucosa (P), normal mucosa (N), and feces (F) sites. **(A)** Comparison of α diversity indexes (ACE, Chao1, Shannon, and Simpson) between the FOBT positive and FOBT negative groups at different sampling sites. **(B)** Relative abundance (%) of phyla in the FOBT positive and FOBT negative groups at different sampling sites.

## Abbreviations

CRC: Colorectal cancer; SCFAs: Short-chain fatty acids; FOBT: Fecal occult blood test; FIT: Fecal immunochemical test; BMI: Body mass index; PCR: Polymerase chain reaction; OTUs: Operational taxonomic units; PCoA: Principal coordinates analysis; PERMANOVA: Permutational multivariate analysis of variance; LDA: Linear discriminant analysis; LEfSe: Linear discriminant analysis effect size; SEM: Standard error of the mean; TNM: tumor-node-metastasis; IL: Interleukin; TGF-β: Transforming growth factor β; TNF: Tumor necrosis factor.

## References

[B1] Al-Hassi H. O. Ng O. Brookes M. (2018). Tumour-associated and Non-tumour-associated Microbiota in Colorectal Cancer. Gut 67 (2), 395. 10.1136/gutjnl-2017-314219 28473629

[B2] Amieva M. Peek R. M. (2016). Pathobiology of Helicobacter Pylori-Induced Gastric Cancer. Gastroenterology 150 (1), 64–78. 10.1053/j.gastro.2015.09.004 26385073 PMC4691563

[B3] Arbyn M. Anttila A. Jordan J. Ronco G. Schenck U. Segnan N. (2010). European Guidelines for Quality Assurance in Cervical Cancer Screening. Second Edition-Summary Document. Ann. Oncol. 21 (3), 448–458. 10.1093/annonc/mdp471 20176693 PMC2826099

[B4] Argilés G. Tabernero J. Labianca R. Hochhauser D. Salazar R. Iveson T. (2020). Localised Colon Cancer: ESMO Clinical Practice Guidelines for Diagnosis, Treatment and Follow-Up. Ann. Oncol. 31 (10), 1291–1305. 10.1016/j.annonc.2020.06.022 32702383

[B5] Baxter N. T. Koumpouras C. C. Rogers M. A. M. Ruffin M. T. Schloss P. D. (2016a). DNA from Fecal Immunochemical Test Can Replace Stool for Detection of Colonic Lesions Using a Microbiota-Based Model. Microbiome 4 (1), 59. 10.1186/s40168-016-0205-y 27842559 PMC5109736

[B6] Baxter N. T. Ruffin M. T. Rogers M. A. M. Schloss P. D. (2016b). Microbiota-based Model Improves the Sensitivity of Fecal Immunochemical Test for Detecting Colonic Lesions. Genome Med. 8 (1), 37. 10.1186/s13073-016-0290-3 27056827 PMC4823848

[B7] Castellarin M. Warren R. L. Freeman J. D. Dreolini L. Krzywinski M. Strauss J. (2012). Fusobacterium Nucleatum Infection Is Prevalent in Human Colorectal Carcinoma. Genome Res. 22 (2), 299–306. 10.1101/gr.126516.111 22009989 PMC3266037

[B8] Chen H.-M. Yu Y.-N. Wang J.-L. Lin Y.-W. Kong X. Yang C.-Q. (2013). Decreased Dietary Fiber Intake and Structural Alteration of Gut Microbiota in Patients with Advanced Colorectal Adenoma. Am. J. Clin. Nutr. 97 (5), 1044–1052. 10.3945/ajcn.112.046607 23553152

[B9] Chen S. Zhou Y. Chen Y. Gu J. (2018). Fastp: an Ultra-fast All-In-One FASTQ Preprocessor. Bioinformatics 34 (17), i884–i890. 10.1093/bioinformatics/bty560 30423086 PMC6129281

[B10] Cueva C. Silva M. Pinillos I. Bartolomé B. Moreno-Arribas M. V. (2020). Interplay between Dietary Polyphenols and Oral and Gut Microbiota in the Development of Colorectal Cancer. Nutrients 12 (3), 625. 10.3390/nu12030625 PMC7146370 32120799

[B11] Dejea C. M. Wick E. C. Hechenbleikner E. M. White J. R. Mark Welch J. L. Rossetti B. J. (2014). Microbiota Organization Is a Distinct Feature of Proximal Colorectal Cancers. Proc. Natl. Acad. Sci. U.S.A. 111 (51), 18321–18326. 10.1073/pnas.1406199111 25489084 PMC4280621

[B12] Deshpande N. P. Wilkins M. R. Castaño-Rodríguez N. Bainbridge E. Sodhi N. Riordan S. M. (2016). Campylobacter Concisus Pathotypes Induce Distinct Global Responses in Intestinal Epithelial Cells. Sci. Rep. 6, 34288. 10.1038/srep34288 27677841 PMC5039708

[B13] Donohoe D. R. Holley D. Collins L. B. Montgomery S. A. Whitmore A. C. Hillhouse A. (2014). A Gnotobiotic Mouse Model Demonstrates that Dietary Fiber Protects against Colorectal Tumorigenesis in a Microbiota- and Butyrate-dependent Manner. Cancer Discov. 4 (12), 1387–1397. 10.1158/2159-8290.CD-14-0501 25266735 PMC4258155

[B14] Edgar R. C. (2013). UPARSE: Highly Accurate OTU Sequences from Microbial Amplicon Reads. Nat. Methods 10 (10), 996–998. 10.1038/nmeth.2604 23955772

[B15] Flemer B. Lynch D. B. Brown J. M. R. Jeffery I. B. Ryan F. J. Claesson M. J. (2017). Tumour-associated and Non-tumour-associated Microbiota in Colorectal Cancer. Gut 66 (4), 633–643. 10.1136/gutjnl-2015-309595 26992426 PMC5529966

[B16] Gao Z. Guo B. Gao R. Zhu Q. Qin H. (2015). Microbiota Disbiosis Is Associated with Colorectal Cancer. Front. Microbiol. 6, 20. 10.3389/fmicb.2015.00020 25699023 PMC4313696

[B17] Hardcastle J. D. Chamberlain J. O. Robinson M. H. Moss S. M. Amar S. S. Balfour T. W. (1996). Randomised Controlled Trial of Faecal-Occult-Blood Screening for Colorectal Cancer. Lancet 348 (9040), 1472–1477. 10.1016/S0140-6736(96)03386-7 8942775

[B18] Jalanka J. Salonen A. Salojärvi J. Ritari J. Immonen O. Marciani L. (2015). Effects of Bowel Cleansing on the Intestinal Microbiota. Gut 64 (10), 1562–1568. 10.1136/gutjnl-2014-307240 25527456

[B19] Kaplan C. W. Lux R. Haake S. K. Shi W. (2009). The Fusobacterium Nucleatumouter Membrane Protein RadD Is an Arginine-Inhibitable Adhesin Required for Inter-species Adherence and the Structured Architecture of Multispecies Biofilm. Mol. Microbiol. 71 (1), 35–47. 10.1111/j.1365-2958.2008.06503.x 19007407 PMC2741168

[B20] Kaplan C. W. Ma X. Paranjpe A. Jewett A. Lux R. Kinder-Haake S. (2010). Fusobacterium Nucleatum Outer Membrane Proteins Fap2 and RadD Induce Cell Death in Human Lymphocytes. Infect. Immun. 78 (11), 4773–4778. 10.1128/IAI.00567-10 20823215 PMC2976331

[B21] Kerr J. Anderson C. Lippman S. M. (2017). Physical Activity, Sedentary Behaviour, Diet, and Cancer: an Update and Emerging New Evidence. Lancet Oncol. 18 (8), E457–E471. 10.1016/s1470-2045(17)30411-4 28759385 PMC10441558

[B22] Kewenter J. Brevinge H. Engarás B. Haglind E. Ährén C. (1994). Results of Screening, Rescreening, and Follow-Up in a Prospective Randomized Study for Detection of Colorectal Cancer by Fecal Occult Blood Testing: Results for 68,308 Subjects. Scand. J. Gastroenterol. 29 (5), 468–473. 10.3109/00365529409096840 8036464

[B23] Koliarakis I. Messaritakis I. Nikolouzakis T. K. Hamilos G. Souglakos J. Tsiaoussis J. (2019). Oral Bacteria and Intestinal Dysbiosis in Colorectal Cancer. Ijms 20 (17), 4146. 10.3390/ijms20174146 PMC6747549 31450675

[B24] Kostic A. D. Gevers D. Pedamallu C. S. Michaud M. Duke F. Earl A. M. (2012). Genomic Analysis Identifies Association of Fusobacterium with Colorectal Carcinoma. Genome Res. 22 (2), 292–298. 10.1101/gr.126573.111 22009990 PMC3266036

[B25] Kostic A. D. Chun E. Robertson L. Glickman J. N. Gallini C. A. Michaud M. (2013). Fusobacterium Nucleatum Potentiates Intestinal Tumorigenesis and Modulates the Tumor-Immune Microenvironment. Cell Host Microbe 14 (2), 207–215. 10.1016/j.chom.2013.07.007 23954159 PMC3772512

[B26] Li S. Konstantinov S. R. Smits R. Peppelenbosch M. P. (2017). Bacterial Biofilms in Colorectal Cancer Initiation and Progression. Trends Mol. Med. 23 (1), 18–30. 10.1016/j.molmed.2016.11.004 27986421

[B27] Liang Q. Chiu J. Chen Y. Huang Y. Higashimori A. Fang J. (2017). Fecal Bacteria Act as Novel Biomarkers for Noninvasive Diagnosis of Colorectal Cancer. Clin. Cancer Res. 23 (8), 2061–2070. 10.1158/1078-0432.Ccr-16-1599 27697996

[B28] Long X. Wong C. C. Tong L. Chu E. S. H. Ho Szeto C. Go M. Y. Y. (2019). Peptostreptococcus Anaerobius Promotes Colorectal Carcinogenesis and Modulates Tumour Immunity. Nat. Microbiol. 4 (12), 2319–2330. 10.1038/s41564-019-0541-3 31501538

[B29] Louis P. Hold G. L. Flint H. J. (2014). The Gut Microbiota, Bacterial Metabolites and Colorectal Cancer. Nat. Rev. Microbiol. 12 (10), 661–672. 10.1038/nrmicro3344 25198138

[B30] Magoc T. Salzberg S. L. (2011). FLASH: Fast Length Adjustment of Short Reads to Improve Genome Assemblies. Bioinformatics 27 (21), 2957–2963. 10.1093/bioinformatics/btr507 21903629 PMC3198573

[B31] Mandel J. S. Bond J. H. Church T. R. Snover D. C. Bradley G. M. Schuman L. M. (1993). Reducing Mortality from Colorectal Cancer by Screening for Fecal Occult Blood. Minnesota Colon Cancer Control Study. N. Engl. J. Med. 328 (19), 1365–1371. 10.1056/NEJM199305133281901 8474513

[B32] McCoy A. N. Araújo-Pérez F. Azcárate-Peril A. Yeh J. J. Sandler R. S. Keku T. O. (2013). Fusobacterium Is Associated with Colorectal Adenomas. PLoS One 8 (1), e53653. 10.1371/journal.pone.0053653 23335968 PMC3546075

[B33] Mira-Pascual L. Cabrera-Rubio R. Ocon S. Costales P. Parra A. Suarez A. (2015). Microbial Mucosal Colonic Shifts Associated with the Development of Colorectal Cancer Reveal the Presence of Different Bacterial and Archaeal Biomarkers. J. Gastroenterol. 50 (2), 167–179. 10.1007/s00535-014-0963-x 24811328

[B34] Nakajima M. Arimatsu K. Kato T. Matsuda Y. Minagawa T. Takahashi N. (2015). Oral Administration of P. Gingivalis Induces Dysbiosis of Gut Microbiota and Impaired Barrier Function Leading to Dissemination of Enterobacteria to the Liver. PLoS One 10 (7), e0134234. 10.1371/journal.pone.0134234 26218067 PMC4517782

[B35] Nakatsu G. Li X. Zhou H. Sheng J. Wong S. H. Wu W. K. K. (2015). Gut Mucosal Microbiome across Stages of Colorectal Carcinogenesis. Nat. Commun. 6, 8727. 10.1038/ncomms9727 26515465 PMC4640069

[B36] Okuda S. Shimada Y. Tajima Y. Yuza K. Hirose Y. Ichikawa H. (2021). Profiling of Host Genetic Alterations and Intra-tumor Microbiomes in Colorectal Cancer. Comput. Struct. Biotechnol. J. 19, 3330–3338. 10.1016/j.csbj.2021.05.049 34188781 PMC8202188

[B37] Osman M. A. Neoh H.-m. Ab Mutalib N.-S. Chin S.-F. Mazlan L. Raja Ali R. A. (2021). Parvimonas Micra, Peptostreptococcus Stomatis, Fusobacterium Nucleatum and Akkermansia Muciniphila as a Four-Bacteria Biomarker Panel of Colorectal Cancer. Sci. Rep. 11 (1), 2925. 10.1038/s41598-021-82465-0 33536501 PMC7859180

[B38] Parahitiyawa N. B. Jin L. J. Leung W. K. Yam W. C. Samaranayake L. P. (2009). Microbiology of Odontogenic Bacteremia: beyond Endocarditis. Clin. Microbiol. Rev. 22 (1), 46–64. 10.1128/CMR.00028-08 19136433 PMC2620633

[B39] Phipps O. Quraishi M. N. Dickson E. A. Steed H. Kumar A. Acheson A. G. (2021). Differences in the On- and Off-Tumor Microbiota between Right- and Left-Sided Colorectal Cancer. Microorganisms 9 (5), 1108. 10.3390/microorganisms9051108 34065545 PMC8160982

[B40] Rubinstein M. R. Wang X. Liu W. Hao Y. Cai G. Han Y. W. (2013). Fusobacterium Nucleatum Promotes Colorectal Carcinogenesis by Modulating E-Cadherin/β-Catenin Signaling via its FadA Adhesin. Cell Host Microbe 14 (2), 195–206. 10.1016/j.chom.2013.07.012 23954158 PMC3770529

[B41] Saito T. Nishikawa H. Wada H. Nagano Y. Sugiyama D. Atarashi K. (2016). Two FOXP3(+)CD4(+) T Cell Subpopulations Distinctly Control the Prognosis of Colorectal Cancers. Nat. Med. 22 (6), 679–684. 10.1038/nm.4086 27111280

[B42] Segata N. Haake S. Mannon P. Lemon K. P. Waldron L. Gevers D. (2012). Composition of the Adult Digestive Tract Bacterial Microbiome Based on Seven Mouth Surfaces, Tonsils, Throat and Stool Samples. Genome Biol. 13 (6), R42. 10.1186/gb-2012-13-6-r42 22698087 PMC3446314

[B43] Strauss J. Kaplan G. G. Beck P. L. Rioux K. Panaccione R. Devinney R. (2011). Invasive Potential of Gut Mucosa-Derived Fusobacterium Nucleatum Positively Correlates with IBD Status of the Host. Inflamm. Bowel Dis. 17 (9), 1971–1978. 10.1002/ibd.21606 21830275

[B44] Sung H. Ferlay J. Siegel R. L. Laversanne M. Soerjomataram I. Jemal A. (2021). Global Cancer Statistics 2020: GLOBOCAN Estimates of Incidence and Mortality Worldwide for 36 Cancers in 185 Countries. CA A Cancer J. Clin. 71 (3), 209–249. 10.3322/caac.21660 33538338

[B45] Tilg H. Adolph T. E. Gerner R. R. Moschen A. R. (2018). The Intestinal Microbiota in Colorectal Cancer. Cancer Cell 33 (6), 954–964. 10.1016/j.ccell.2018.03.004 29657127

[B46] Wang Q. Garrity G. M. Tiedje J. M. Cole J. R. (2007). Naïve Bayesian Classifier for Rapid Assignment of rRNA Sequences into the New Bacterial Taxonomy. Appl. Environ. Microbiol. 73 (16), 5261–5267. 10.1128/AEM.00062-07 17586664 PMC1950982

[B47] Warren R. L. Freeman D. J. Pleasance S. Watson P. Moore R. A. Cochrane K. (2013). Co-occurrence of Anaerobic Bacteria in Colorectal Carcinomas. Microbiome 1 (1), 16. 10.1186/2049-2618-1-16 24450771 PMC3971631

[B48] Wirbel J. Pyl P. T. Kartal E. Zych K. Kashani A. Milanese A. (2019). Meta-analysis of Fecal Metagenomes Reveals Global Microbial Signatures that Are Specific for Colorectal Cancer. Nat. Med. 25 (4), 679–689. 10.1038/s41591-019-0406-6 30936547 PMC7984229

[B49] Wu J. Li Q. Fu X. (2019). Fusobacterium Nucleatum Contributes to the Carcinogenesis of Colorectal Cancer by Inducing Inflammation and Suppressing Host Immunity. Transl. Oncol. 12 (6), 846–851. 10.1016/j.tranon.2019.03.003 30986689 PMC6462820

[B50] Yachida S. Mizutani S. Shiroma H. Shiba S. Nakajima T. Sakamoto T. (2019). Metagenomic and Metabolomic Analyses Reveal Distinct Stage-specific Phenotypes of the Gut Microbiota in Colorectal Cancer. Nat. Med. 25 (6), 968–976. 10.1038/s41591-019-0458-7 31171880

[B51] Yuan B. Ma B. Yu J. Meng Q. Du T. Li H. (2021). Fecal Bacteria as Non-invasive Biomarkers for Colorectal Adenocarcinoma. Front. Oncol. 11, 664321. 10.3389/fonc.2021.664321 34447694 PMC8383742

[B52] Zagato E. Pozzi C. Bertocchi A. Schioppa T. Saccheri F. Guglietta S. (2020). Endogenous Murine Microbiota Member Faecalibaculum Rodentium and its Human Homologue Protect from Intestinal Tumour Growth. Nat. Microbiol. 5 (3), 511–524. 10.1038/s41564-019-0649-5 31988379 PMC7048616

[B53] Zeller G. Tap J. Voigt A. Y. Sunagawa S. Kultima J. R. Costea P. I. (2014). Potential of Fecal Microbiota for Early‐stage Detection of Colorectal Cancer. Mol. Syst. Biol. 10, 766. 10.15252/msb.20145645 25432777 PMC4299606

