# Supplementary figures and images for: Gut Microbiota Signatures in Tumor, Para-Cancerous, Normal Mucosa, and Feces in Colorectal Cancer Patients
Source: Front Cell Dev Biol. 2022 Jun 2;10:916961. doi: 10.3389/fcell.2022.916961 (PMC9201480; doi:10.3389/fcell.2022.916961)

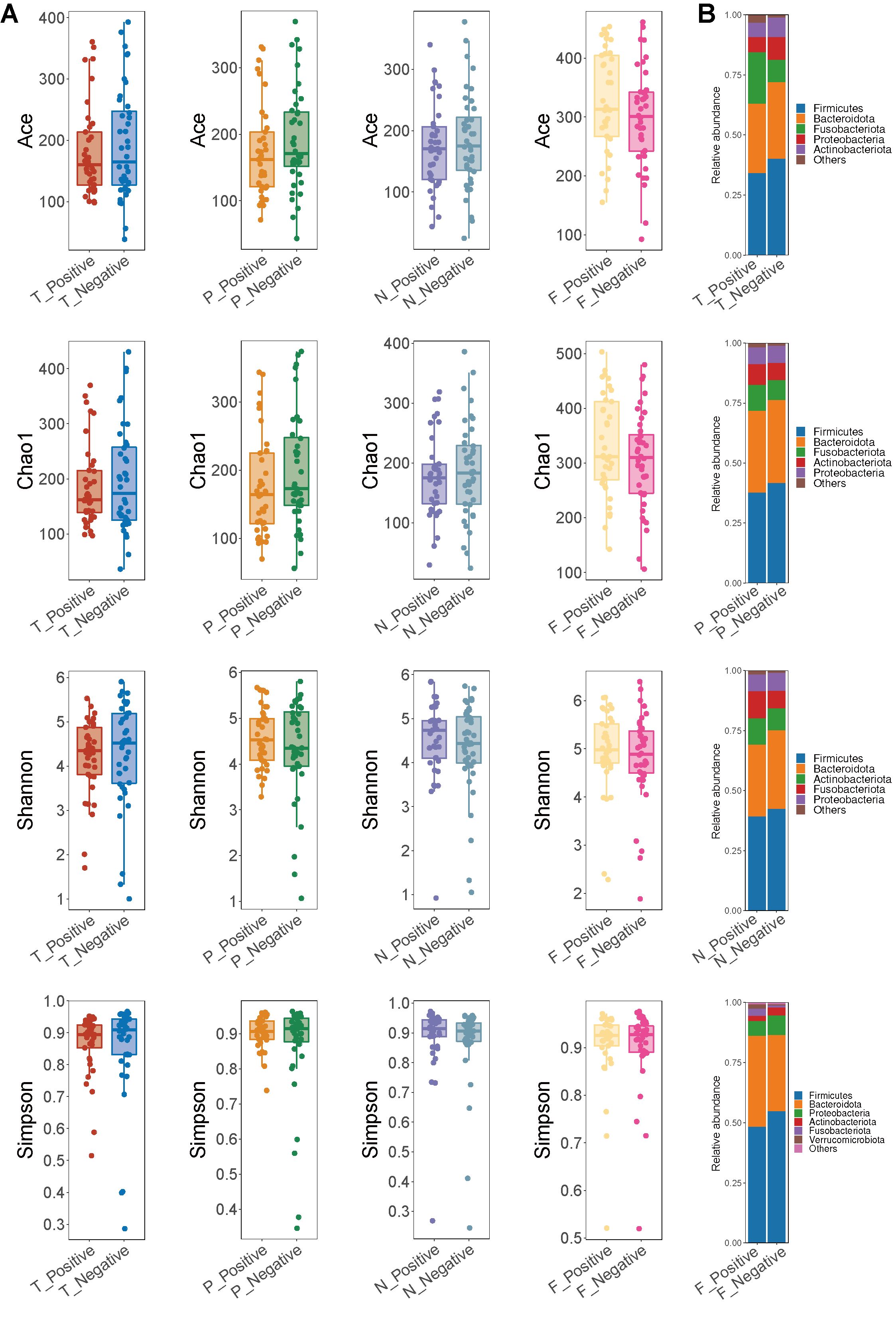

Supplement: Supplementary file 1 [file Image2.tif]

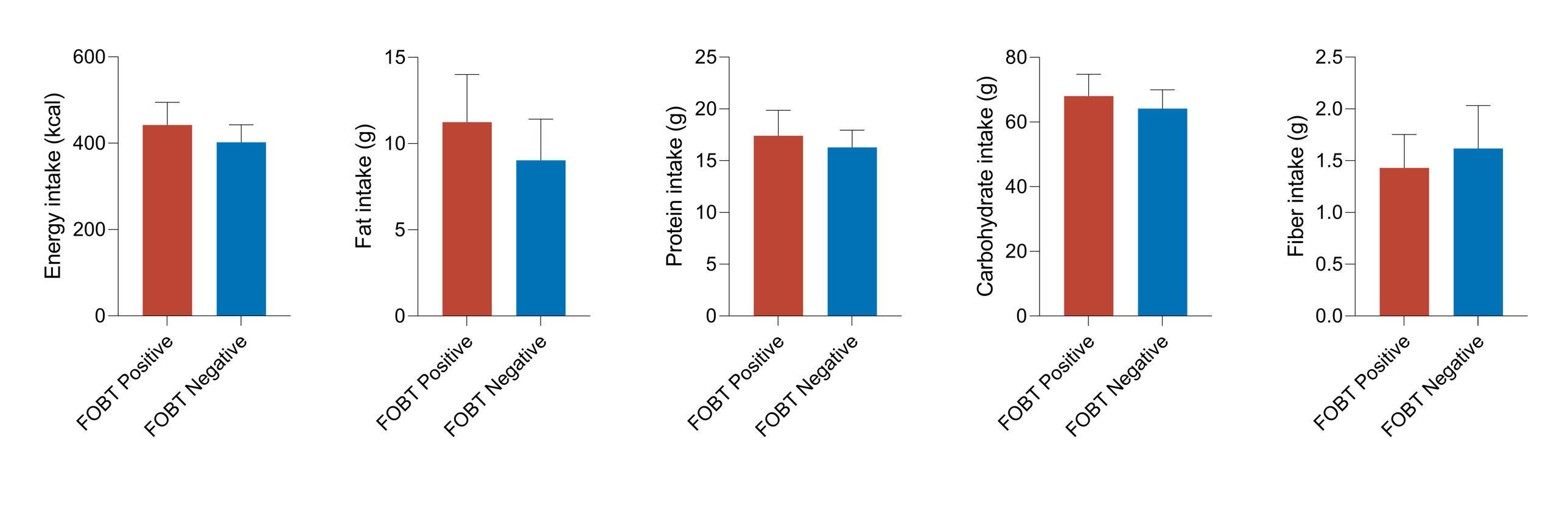

Supplement: Supplementary file 2 [file Image1.tif]
